# Nonlinear association between birth weight and overweight/obesity in children aged 3–6 years: a retrospective analysis of 13 cities in China

**DOI:** 10.3389/fnut.2026.1883738

**Published:** 2026-09-23

**Authors:** Su Liu, Sen Li, Sanhua Zhang, Jian Song, Chunjing Tu, Huiming Huang

**Affiliations:** 1 College of Physical Education and Health, China Pharmaceutical University, Nanjing, China; 2 Jiangsu Provincial Center of National Physical Fitness Monitoring, Nanjing, China; 3 Jing Hengyi School of Education, Hangzhou Normal University, Hangzhou, China; 4 School of Teacher (Physical) Education, Taizhou University, Taizhou, China

**Keywords:** birth weight, children, nonlinear, obesity, overweight

## Abstract

**Objective:**

The aim of this study was to explore the impact of birth weight on overweight and obesity in children aged 3–6 years across 13 cities in China, as well as the potential interactions.

**Methods:**

This study was designed as a retrospective analysis adhering to the STROBE guidelines. Data were derived from three waves of the Physical Fitness Survey in Jiangsu targeting children aged 3–6 years, which employed a multi-stage, stratified, cluster probability sampling design to obtain a representative sample from thirteen cities. Overweight and obesity were defined according to the Chinese National Health Industry Standard WS/T 423—2022 (issued in 2022) based on BMI standard deviation scores. Eight covariates, including age, sex, survey year, geographic region, urban–rural status, gestational age, childbearing age, and mother’s education, were included. Multivariate logistic regression was used to estimate odds ratios (ORs) and 95% confidence intervals (CIs) for the risks of overweight, obesity and overweight/obesity associated with low birth weight and macrosomia after adjusting for covariates. Subgroup analysis results were visualized using forest plots; restricted cubic spline (RCS) analysis was performed to examine nonlinear and dose–response relationships; and sensitivity analysis included mixed-effects logistic regression models to adjust for clustering effects, changing birth weight classification cut-points, and excluding gestational age (a key potential covariate).

**Results:**

A total of 23,362 participants were included. The prevalence of overweight, obesity, and overweight/obesity was 19.2, 9, and 28.2%, respectively. (1) Birth weight exhibited a nonlinear association with weight and BMI at 3–6 years, with a gentle slope in the early segment and a steeper slope in the later segment (P for nonlinearity < 0.001). (2) After covariate adjustment, low birth weight was not associated with overweight risk (*p* = 0.165), although it was associated with obesity and combined overweight/obesity risks (OR = 0.628 and 0.736, *p* = 0.027 and 0.010). However, after stratification by gender, age, geography, and urban–rural status, all subgroups showed no statistical significance; gestational age had a significant interaction with low birth weight, with low birth weight (term birth) having lower overweight/obesity risk (*p* = 0.001), while preterm infants showed no overweight/obesity risk (*p* > 0.05). (3)Macrosomia was significantly associated with increased risks of overweight (OR = 1.464, 95%CI: 1.326–1.616), obesity (OR = 2.346, 95%CI: 2.087–2.648), and overweight/obesity (OR = 2.006, 95%CI: 1.839–2.188) compared with normal birth weight (all *p* < 0.001); the subgroup analysis indicated a more pronounced trend in younger age groups (*p* < 0.001). (4) After covariate adjustment, the relationship between birth weight and risks of obesity/overweight/obesity was flat in the lower range and increased sharply in the higher range, demonstrating a nonlinear dose–response relationship (P for nonlinearity < 0.001).

**Conclusion:**

Birth weight shows nonlinear associations with weight, BMI, and risks of obesity/overweight/obesity in children aged 3–6 years, characterized by a gentle slope in the lower birth weight range and a steeper slope in the higher range. Low birth weight has limited impact on the development of overweight and obesity, but gestational age shows significant heterogeneity; whereas macrosomia confers significantly higher risks of overweight, obesity, and overweight/obesity compared with normal birth weight, with this effect diminishing with increasing age.

## 1 Introduction

In recent years, the prevalence of overweight and obesity has risen sharply, affecting not only adults but also children (1, 2). Overweight/obesity resulting from childhood overweight and obesity is currently one of the most serious public health concerns. It is projected that by 2035, the prevalence of overweight and obesity among Chinese children and adolescents will reach 41.9% (3). This has raised concerns among public health practitioners, as childhood obesity increases the risk of adult obesity (4). Childhood obesity not only impairs physical and mental health but also poses severe long-term health risks, such as the development of diabetes and cardiovascular diseases, and often persists into adulthood (5–7). According to the Developmental Origins of Health and Disease (DOHaD) hypothesis (8), researchers have focused on the link between fetal origins and health. Fetal nutritional status is one of the factors influencing birth weight, and an adverse intrauterine environment is more likely to lead to postnatal obesity (9). Birth weight may be a key indicator of fetal nutritional status, and studies have shown that its effects may persist into later health outcomes (10–12). Thus, ensuring optimal birth weight and monitoring/controlling childhood overweight/obesity (overweight or obesity) may be the first step in the primary prevention of childhood obesity.

Epidemiological studies have indicated a direct relationship between birth weight and childhood BMI/obesity: research from Brazil (13), China (14–17), Turkey (18), Uruguay (19), and Poland (20) show that higher birth weight increases the risk of childhood obesity. An earlier review noted that a positive correlation between birth weight and childhood obesity predominates in the published literature (21). In contrast, a retrospective cohort study in Brazil found no association between birth weight and obesity in children aged 7–14 years (22); a small-sample study in Poland also reported no difference in birth weight between overweight/obese children and normal-weight children (10). Additionally, some studies have indicated that high birth weight (but not low birth weight) is associated with increased overweight/obesity risk in childhood (23–26), suggesting a potential nonlinear relationship between birth weight and BMI/overweight/obesity risk.

Inconsistencies in previous studies may be attributed to differences in sample size, ethnicity, and methods of assessing overweight/obesity; limited information on potential confounders (e.g., gestational age, which affects birth weight) may also restrict results (21). Furthermore, few studies have used intuitive restricted cubic spline (RCS) analysis to demonstrate the nonlinear relationship between birth weight and childhood BMI/overweight/obesity. Shi J et al. analyzed the nonlinear relationship between birth weight and overweight/obesity in children aged 7–17 years (26), but the nonlinear relationship in younger children (before 6 years) remains unclear. Researchers may pay greater attention to the impact of birth weight on early childhood weight, BMI, and overweight/obesity, as studies have suggested that the strength of this association tends to decline with advancing age (20). Besides age interaction, some studies have also indicated that gender plays a moderating role (24, 26). However, beyond age and sex, few additional potential interaction factors have been reported previously. Studies show that obesity is also influenced by period effects, particularly during periods with significant social forces (27). Analyzing the moderating effects of these subgroups (such as period, urban–rural status, geographical factors) can provide context-specific recommendations for practical applications.

Therefore, additional studies with larger sample sizes, covering a broader age range of children, and incorporating subgroup analyses of more demographic characteristics to analyze the relationship between birth weight and BMI, overweight, and obesity are needed. This study aimed to quantify the associations between birth weight and weight, BMI, and risks of overweight, obesity, and overweight/obesity in children aged 3–6 years using data from 13 cities in Jiangsu Province covered by the National Physical Fitness Surveillance, and to analyze whether subgroup factors (child age, sex, survey year, geographic region, and urban–rural status) have modifying effects.

## 2 Materials and methods

### 2.1 Study design

The study design is a retrospective analysis, where the independent variables, covariates, and outcomes follow a temporal sequence. The research methods comply with the STROBE Statement (Strengthening the Reporting of Observational Studies in Epidemiology) (28), which should be adhered to in observational studies.

### 2.2 Participants and data collection

We utilized data on children aged 3–6 years from three waves (2011, 2015, and 2019) of the Physical Fitness Survey in Jiangsu (PFSJ), which encompasses thirteen cities (Nanjing, Suzhou, Wuxi, Changzhou, Nantong, Zhenjiang, Yangzhou, Taizhou, Xuzhou, Huaian, Suqian, Lianyungang, and Yancheng). As a program initiated by the government sports department, PFSJ demonstrates high representativeness of the general population, encompassing 85.26 million permanent residents across 13 cities in Jiangsu Province. The survey employed a multi-stage, stratified, cluster probability sampling design to draw a representative sample from these 13 cities. The first stage encompassed all 13 prefecture-level cities in Jiangsu Province. In the second stage, two districts (representing urban areas) and two counties (representing rural areas) were selected from each city. The third stage involved randomly selecting two sub-districts from each urban district and two towns from each rural county. During the fourth stage, two residential communities were chosen from each sub-district and two administrative villages from each town. In the final stage, kindergartens were selected using systematic sampling, ensuring a minimum of 35 children per single-year age group from 3 to 6 years in each kindergarten.

All procedures underwent quality control by field supervisors before data were submitted to the Jiangsu Provincial Physical Fitness Monitoring Center. Written informed consent was obtained from the parents of each participating child. The overall study protocol of this secondary dataset analysis was approved by the Ethics Committee of the Faculty of Sports Science at Ningbo University (Approval No. TY 2025046). PFSJ data comprise physical fitness tests and questionnaire surveys. A total of 23,362 participants were effectively included in the final sample (7,658 in 2011, 7,008 in 2015, and 8,696 in 2019). The flow chart of participant selection and sample size distribution chart for 13 cities in Jiangsu Province are presented in Figure 1.

**Figure 1:**
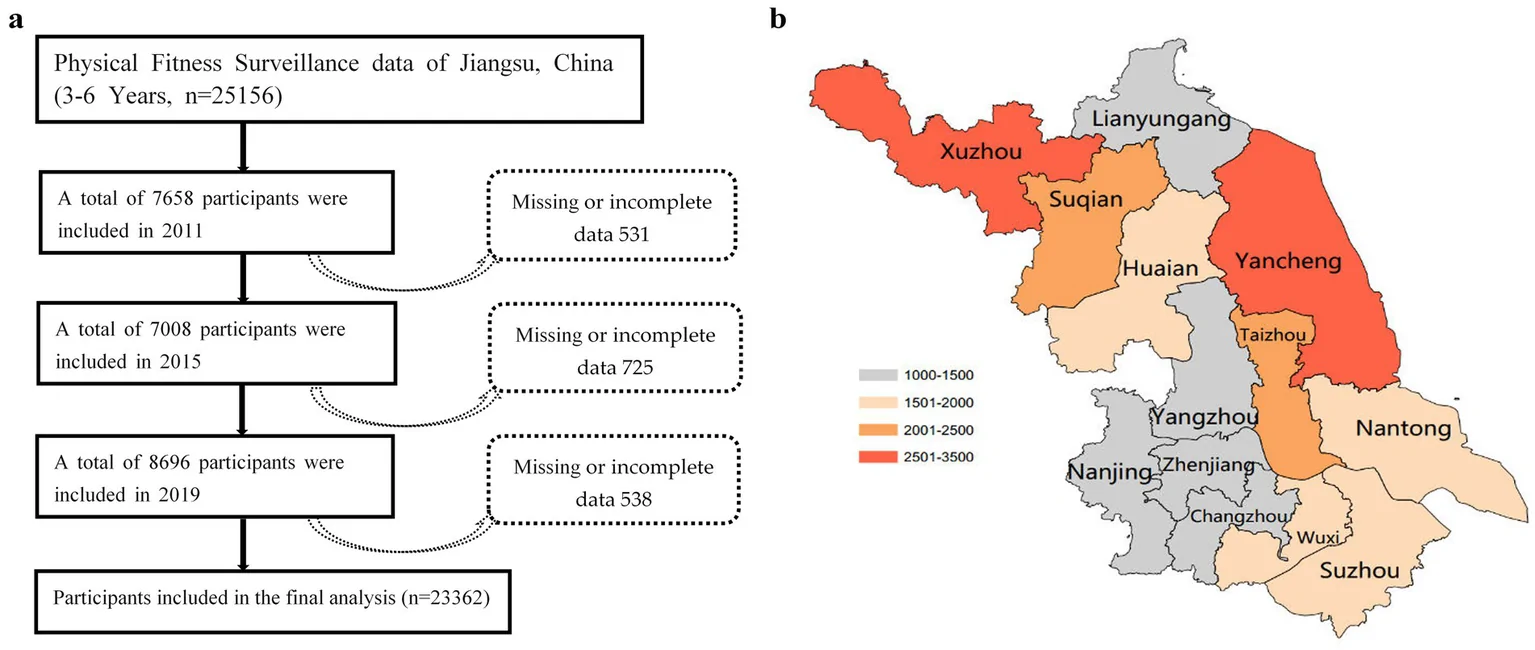
Flow chart **(a)** of the selection of participants and sample size distribution chart **(b)** for 13 cities in Jiangsu Province.

### 2.3 Measurements and definition

#### 2.3.1 Outcomes and dependent variables

Height was measured using a stadiometer with participants standing barefoot. Body weight was measured using a standardized electronic scale. Height and weight were recorded to the nearest 0.1 cm and 0.1 kg, respectively. Body mass index (BMI) was calculated as weight in kilograms divided by height in meters squared (kg/m^2^). All measurements were conducted in accordance with the National Student Physical Health Standard (29).

Definition: Overweight and obesity were defined based on the Chinese National Health Industry Standard WS/T 423—2022 issued in 2022, which establishes authoritative BMI-for-age or weight-for-height standard deviation (SD) classifications for assessing nutritional status in Chinese children aged 0–7 years (30). In this study, the BMI-for-age classification was adopted, which accounts for age, weight, and height. The criteria are as follows: overweight: +1 SD ≤ BMI < +2 SD; obesity: +2 SD ≤ BMI < +3 SD; severe obesity: BMI ≥ +3 SD. Children with severe obesity were merged into the obesity category. The term “overweight/obesity” refers to the combined group of overweight and obesity.

#### 2.3.2 Independent variables and covariates

Birth weight, the independent variable in this study, was reported by the children’s parents. Parents were instructed to record birth weight (in kilograms, accurate to one decimal place) with reference to birth certificates. Infants with a birth weight < 2.5 kg were defined as low birth weight; those with a birth weight ≥ 4.0 kg were classified as high birth weight or macrosomia; birth weight ≥ 2.5 kg and < 4.0 kg was categorized as normal (31).

Child-related demographic characteristics and maternal-related factors were considered as covariates. These included age (continuous variable), survey year (2011, 2015, 2019), sex (male, female), geographic region (southern Jiangsu, central Jiangsu, northern Jiangsu, based on geographic and administrative divisions: southern Jiangsu includes Nanjing, Suzhou, Wuxi, Zhenjiang, and Changzhou; central Jiangsu includes Yangzhou, Taizhou, and Nantong; northern Jiangsu includes Xuzhou, Huaian, Suqian, Lianyungang, and Yancheng), urban–rural status (urban, rural), gestational age (recorded from birth certificate; preterm birth: ≤36 weeks, term birth: 37–41 weeks, post-term birth: ≥42 weeks), childbearing age (<25 years, 25–29 years, ≥30 years) (31), and mother’s education level (low: below high school; medium: high school or vocational school; high: college or above).

### 2.4 Statistical analysis

Categorical variables were reported as frequencies with proportions, and continuous variables were reported as means. The means and prevalence of categorical variables were compared using analysis of variance (ANOVA) and χ^2^ tests, respectively. ZstatsMultivariate logistic regression was used to estimate odds ratios (ORs) and 95% confidence intervals (CIs) for the risks of overweight, obesity, and overweight/obesity associated with low birth weight and macrosomia after adjusting for covariates. We used restricted cubic splines (RCS) to analyze the nonlinear relationships between birth weight and the outcomes (weight, BMI, and risk of overweight/obesity at 3–6 years of age) after adjusting for covariates, with five knots (5, 27.5, 50, 72.5, 95%) selected based on the large sample size. Subgroup analyses were conducted based on children’s demographic characteristics (age category, survey year, sex, geographic region, and urban–rural status).

We employed three sensitivity analysis methods to verify the reliability and robustness of the results: (1) Changing statistical methods. Due to potential clustering effects from different kindergartens that might influence logistic regression, we used 13 cities as random intercept variance to conduct mixed-effects logistic regression models to adjust for clustering effects and compared the results with standard logistic regression. (2) Re-categorizing birth weight using alternative cutoffs, low birth weight defined as < 2.6 kg and macrosomia as ≥ 3.9 kg; (3) adjusting for different sets of core covariates to assess the robustness of the results. Given the influence of gestational age on birth weight, models were fitted both with and without adjustment for gestational age (see Appendix Tables A1–A4). Statistical significance was set at a two-tailed *p*-value < 0.05. Data processing and analysis were performed using SPSS27.0 and R version 4.3.0, along with Zstats l.0.^1^

## 3 Results

### 3.1 Participants’ characteristics and univariate analysis

A total of 23,362 valid samples were included, of which males comprised 51.1% and females comprised 48.9%. The mean age of subjects was 4.91 years (standard deviation 1.04 years), with a median age of 4.92 years (interquartile range 4.00–5.83 years). The mean birth weight, weight at 3–6 years, and BMI were 3.43 kg, 19.89 kg, and 16.08, respectively. Missing data for some covariates: gestational age missing for 10 participants, maternal age at childbirth missing for 46 participants, maternal education missing for 12 participants; we used SPSS missing variable exclusion method.

The univariate analysis (Table 1) showed that children aged 3–6 years in higher birth weight categories, male, more recent survey years, older age groups, urban children, northern geographic regions, higher maternal reproductive age, and higher maternal education levels had significantly higher weight and BMI (all *p* < 0.001). Additionally, male gender, more recent survey years, northern geographic regions, higher gestational age, and higher maternal reproductive age were associated with higher birth weight (all *p* < 0.001).

**Table 1:** Baseline characteristics and the differences in birthweight, weight, and BMI.

Variables	No. (%)	Birthweight (kg)	*p*	Weight (kg)	*p*	BMI	*p*
Total	23,362 (100)	3.43 (0.46)	-	19.89 (4.24)	-	16.08 (2.48)	-
Birthweight categories			<0.001		<0.001		<0.001
Low	475 (2.0)	2.20 (0.23)		18.86 (4.06)		15.59 (1.73)	
Normal	20,334 (87.1)	3.36 (0.34)		19.63 (4.13)		16.00 (2.47)	
Macrosomia	2,553 (10.9)	4.22 (0.26)		22.08 (4.48)		16.82 (2.46)	
Survey year			0.010		<0.001		<0.001
2011	7,658 (32.8)	3.42 (0.46)		19.40 (3.74)		15.90 (1.64)	
2015	7,008 (30.0)	3.43 (0.46)		20.01 (4.33)		16.02 (2.23)	
2019	8,696 (37.2)	3.44 (0.46)		20.20 (4.53)		16.28 (3.12)	
Sex			<0.001		<0.001		<0.001
Male	11,932 (51.1)	3.48 (0.46)		20.42 (4.43)		16.28 (2.46)	
Female	11,430 (48.9)	3.38 (0.45)		19.33 (3.96)		15.88 (2.47)	
Age at survey			<0.001		<0.001		<0.001
3 years	5,823 (24.9)	3.41 (0.45)		17.11 (3.01)		16.26 (2.70)	
4 years	6,180 (26.5)	3.42 (0.45)		18.56 (3.11)		15.95 (2.27)	
5 years	6,158 (26.4)	3.45 (0.46)		21.05 (3.74)		16.02 (2.45)	
6 years	5,201 (22.3)	3.44 (0.47)		23.20 (4.39)		16.10 (2.46)	
Urban–rural status			0.320		<0.001		<0.001
Urban	10,935 (46.8)	3.43 (0.46)		20.37 (4.44)		16.25 (2.66)	
Rural	12,427 (53.2)	3.43 (0.46)		19.34 (3.94)		15.89 (2.23)	
Geographical region			<0.001		<0.001		<0.001
Southern	6,428 (27.5)	3.39 (0.45)		19.48 (4.08)		15.83 (2.03)	
Central	5,421 (23.2)	3.42 (0.45)		19.84 (4.13)		15.95 (2.01)	
Northern	11,513 (49.3)	3.46 (0.47)		20.13 (4.36)		16.29 (2.85)	
Gestational age			<0.001		0.264		0.095
Preterm birth	1,539 (6.6)	2.94 (0.53)		19.74 (4.36)		16.21 (3.01)	
Term birth	20,481 (87.7)	3.46 (0.43)		19.89 (4.23)		16.07 (2.44)	
Post-term birth	1,332 (5.7)	3.60 (0.46)		19.99 (4.29)		16.12 (2.36)	
Childbearing age			<0.001		<0.001		0.006
< 25 years	11,001 (47.1)	3.42 (0.46)		19.74 (4.22)		16.03 (2.39)	
25–29 years	9,233 (39.5)	3.44 (0.45)		20.01 (4.23)		16.11 (2.45)	
≥ 30 years	3,082 (13.2)	3.47 (0.49)		20.02 (4.34)		16.19 (2.84)	
Mother’s education			0.675		<0.001		<0.001
Low	1,081 (4.6)	3.44 (0.49)		19.45 (3.66)		15.78 (1.77)	
Medium	7,602 (32.5)	3.43 (0.46)		19.63 (4.03)		15.94 (2.30)	
High	14,667 (62.8)	3.43 (0.46)		20.05 (4.38)		16.18 (2.60)	

Values are expressed as mean (SD), and statistical analysis as between-group variance analysis.

### 3.2 Nonlinear trends between birth weight and weight/BMI at 3–6 years

To visually illustrate this relationship, RCS analysis was used to assess the nonlinear trends between birth weight and weight/BMI. RCS trend analysis with 5 knots, using a birth weight of 3.4 kg as the reference value (Figure 2), revealed a statistically significant overall association between birth weight and weight/BMI after adjusting for covariates (continuous age, sex, survey year, geographic region, urban–rural status, gestational age, childbearing age, and mother’s education; P for overall < 0.001). Meanwhile, the nonlinear relationship was also statistically significant, characterized by a gentle slope in the lower birth weight range and a steeper slope in the higher range, resembling a broken-line pattern. Visually, the inflection point for weight was below 3.4 kg (left of the reference value), while the inflection point for BMI was approximately consistent with the reference value (P for nonlinearity < 0.001).

**Figure 2:**
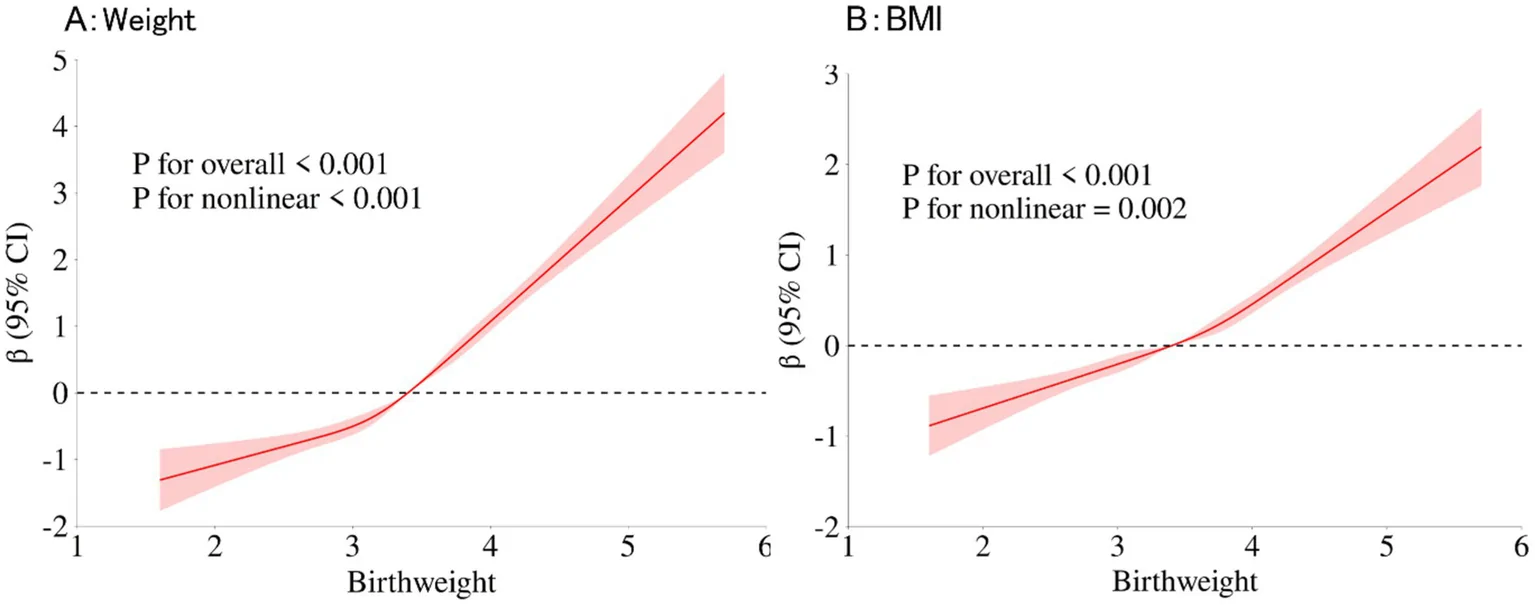
RCS analysis of birth weight with weight and BMI. Shaded areas represent the 95% confidence intervals (95%CI) for regression coefficients. **(A)** Weight. **(B)** BMI.

### 3.3 Prevalence of overweight and obesity across different birth weight categories

Table 2 presents the number of positive cases and prevalence of overweight, obesity, and overweight/obesity across various factors. As shown in Table 2, there were statistically significant differences in the prevalence of overweight, obesity, and overweight/obesity among the three birth weight groups (low-birth-weight, normal, macrosomia; Chi-square test, *p* < 0.001). For both overweight and obesity, the prevalence of overweight/obesity differed substantially between macrosomia and normal birth weight groups; however, the difference narrowed between low birth weight and normal groups, with overweight prevalence of 16.6% vs. 18.6% and overweight/obesity prevalence of 22.3% vs. 26.6%, respectively. Further risk analysis was conducted using multivariate regression.

**Table 2:** Prevalence of overweight and obesity across different birth weight categories.

Variables	Overweight	*p*	Obesity	*p*	Overweight/obesity ^#^	*p*
	No. % (95%CI)		No. % (95%CI)		No. % (95%CI)	
Total	4,479 19.2 (18.7, 19.7)	-	2098 9.0 (8.6, 9.4)	-	6,577 28.2 (27.6, 28.7)	-
Birthweight categories		<0.001		<0.001		<0.001
Low	79 16.6 (13.4, 20.3)		27 5.7 (3.8, 8.2)		106 22.3 (18.6, 26.3)	
Normal	3,773 18.6 (18.0, 19.1)		1,637 8.1 (7.7, 8.4)		5,410 26.6 (26.0, 27.2)	
Macrosomia	627 24.6 (22.9, 26.3)		434 17.0 (15.6, 18.5)		1,061 41.6 (39.6, 43.5)	
Survey year		0.106		<0.001		<0.001
2011	1,506 19.7 (18.8, 20.6)		524 6.8 (6.3, 7.4)		2030 26.5 (25.5, 27.5)	
2015	1,367 19.5 (18.6, 20.5)		603 8.6 (8.0, 9.3)		1970 28.1 (27.1, 29.2)	
2019	1,606 18.5 (17.7, 19.3)		971 11.2 (10.5, 11.8)		2,577 29.6 (28.7, 30.6)	
Sex		0.755		<0.001		<0.001
Male	2,297 19.3 (18.5, 20.0)		1,179 9.9 (9.4, 10.4)		3,476 29.1 (28.3, 30.0)	
Female	2,182 19.1 (18.4, 19.8)		919 8.0 (7.5, 8.6)		3,101 27.1 (26.3, 28.0)	
Age at survey		<0.001		<0.001		<0.001
3 years	1,270 21.8 (20.8, 22.9)		605 10.4 (9.6, 11.2)		1875 32.2 (31.0, 33.4)	
4 years	1,201 19.4 (18.5, 20.4)		492 8.0 (7.3, 8.7)		1,693 27.4 (26.3, 28.5)	
5 years	1,133 18.4 (17.4, 19.4)		554 9.0 (8.3, 9.7)		1,684 27.4 (26.3, 28.5)	
6 years	875 16.8 (15.8, 17.9)		447 8.6 (7.8, 9.4)		1,322 25.4 (24.2, 26.6)	
Urban–rural status		<0.001		<0.001		<0.001
Urban	2,516 20.2 (19.5, 21.0)		1,313 10.6 (10.0, 11.1)		3,829 30.8 (30.0, 31.6)	
Rural	1963 18.0 (17.2, 18.7)		785 7.2 (6.7, 7.7)		2,748 25.1 (24.3, 26.0)	
Geographical region		<0.001		<0.001		<0.001
Southern Jiangsu	1,091 17.0 (16.1, 17.9)		436 6.8 (6.2, 7.4)		1,527 23.8 (22.7, 24.8)	
Central Jiangsu	1,068 19.7 (18.6, 20.8)		440 8.1 (7.4, 8.9)		1,508 27.8 (26.6, 29.0)	
Northern Jiangsu	2,320 20.2 (19.4, 20.9)		1,222 10.6 (10.1, 11.2)		3,542 30.8 (29.9, 31.6)	
Gestational age		0.997		0.217		0.508
Preterm birth	296 19.2 (17.3, 21.2)		141 9.2 (7.7, 10.6)		437 28.4 (26.1, 30.7)	
Term birth	3,925 19.2 (18.6, 19.7)		1820 8.9 (8.5, 9.3)		5,745 28.1 (27.4, 28.7)	
Post-term birth	256 19.2 (17.1, 21.3)		138 10.3 (8.7, 11.9)		393 29.5 (27.1, 31.9)	
Childbearing age		0.531		0.001		0.122
< 25 years	2,105 19.1 (18.4, 19.9)		922 8.4 (7.9, 8.9)		3,027 27.5 (26.7, 28.4)	
25–29 years	1795 19.4 (18.6, 20.3)		849 9.2 (8.6, 9.8)		2,644 28.6 (27.7, 29.6)	
≥ 30 years	571 18.5 (17.2, 19.9)		321 10.4 (9.3, 11.5)		892 28.9 (27.3, 30.5)	
Mother’s education		0.001		<0.001		<0.001
Low	196 18.1 (15.8, 20.4)		71 6.6 (5.1, 8.1)		267 24.7 (22.1, 27.3)	
Medium	1,360 17.9 (17.0, 18.8)		582 7.7 (7.1, 8.3)		1942 25.5 (24.6, 26.5)	
High	2,917 19.9 (19.3, 20.5)		1,442 9.8 (9.4, 10.3)		4,359 29.7 (28.9, 30.5)	

Chi-square tests for univariate proportions.

Furthermore, chi-square tests for univariate proportions showed that more recent survey years, male, younger age, urban children, northern Jiangsu, and higher mother’s education were all associated with higher prevalence of obesity and overweight/obesity (all *p* values <0.001), while univariate analysis of gestational age and childbearing age showed no differences.

### 3.4 Birth weight and the risk of overweight and obesity in children aged 3–6 years

Multivariate logistic regression models estimated the risk of overweight and obesity at 3–6 years by birth weight (with normal birth weight as the reference). Table 3 shows minimal changes between crude and adjusted ORs, *p*-values (adjusted for multiple covariates including continuous age, age, sex, survey year, geographic region, urban–rural status, gestational age, childbearing age, and mother’s education). After covariate adjustment, low birth weight was not associated with increased risks of overweight (OR = 0.834, *p* = 0.165) compared with normal birth weight but was associated with reduced obesity (OR = 0.628, *p* = 0.027) and overweight/obesity risk (OR = 0.736, *p* = 0.010). In contrast, macrosomia was associated with significantly higher overweight and obesity risk: adjusted ORs were 1.464 for overweight (*p* < 0.001), 2.346 for obesity (*p* < 0.001), and 2.006 for overweight/obesity (*p* < 0.001).

**Table 3:** Risks of overweight, obesity, and overweight/obesity associated with low-birth-weight and macrosomia (normal birth weight as reference).

Dependent variable	Low birthweight ^a^		Macrosomia ^a^		Low birthweight ^b^		Macrosomia ^b^	
	OR (95% CI)	*p*	OR (95% CI)	*p*	OR (95% CI)	*p*	OR (95% CI)	*p*
Overweight	0.876 (0.686, 1.118)	0.286	1.429 (1.297, 1.574)	< 0.001	0.834 (0.645, 1.078)	0.165	1.464 (1.326, 1.616)	< 0.001
Obesity	0.688 (0.465, 1.018)	0.062	2.339 (2.085, 2.624)	< 0.001	0.628 (0.421, 0.946)	0.027	2.346 (2.087, 2.648)	< 0.001
Overweight/obesity	0.792 (0.637, 0.986)	0.037	1.962 (1.803, 2.135)	< 0.001	0.736 (0.585, 0.928)	0.010	2.006 (1.839, 2.188)	< 0.001

a Crude model.

b Adjusted for continuous age, sex, survey year, geographic region, urban–rural status, gestational age, childbearing age and mother’s education.

Due to the limited statistical power of the relationship between low birth weight and obesity, overweight/obesity (*p* = 0.027 and 0.010), further subgroup analysis and sensitivity analysis are needed to verify reliability.

Further subgroup analyses (Figure 3 for overweight, Figure 4 for obesity, and Figure 5 for overweight/obesity) evaluated potential interactions with subgroup factors after adjusting for potential confounders (continuous age, sex, survey year, geographic region, urban–rural status, gestational age, childbearing age, and mother’s education). As shown in Figure 3, compared with normal birth weight, except for gestational age (no difference in preterm and post-term births, but term born children had significantly lower overweight risk with interaction *p* value = 0.002), low birth weight was not associated with increased overweight risk in most subgroups (*p* > 0.05), and no significant interactions between subgroups were found (all *p* > 0.05); notably, among low birth weight children we surveyed, preterm births accounted for nearly 60%, and subgroup analysis suggested gestational age was the main source of heterogeneity. The relationship between macrosomia and overweight showed no differences in subgroup analysis (*p* > 0.05).

**Figure 3:**
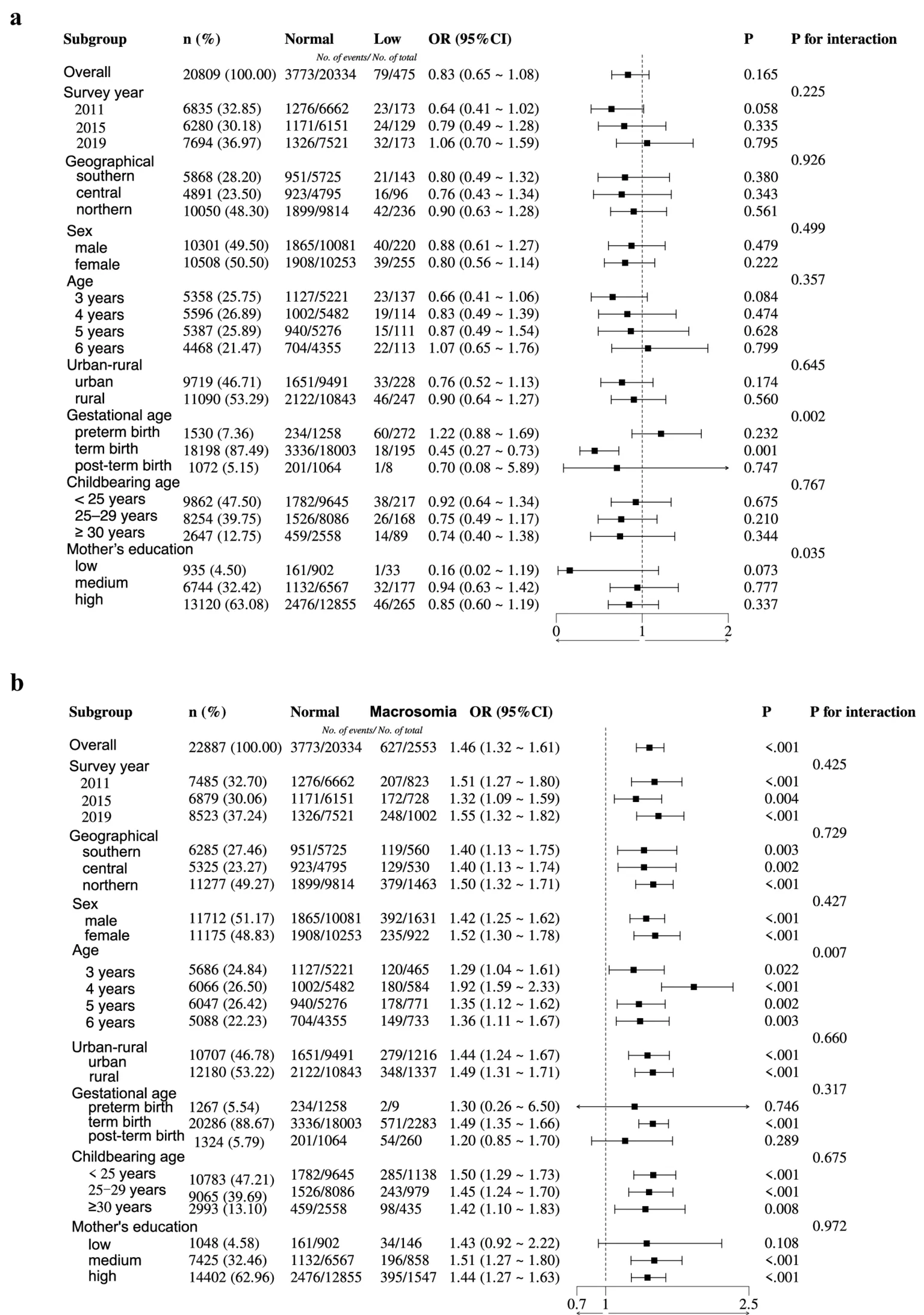
Subgroup analysis of overweight risk for birth weight in children aged 3–6 years. **(a)** normal birthweight vs. low birthweight; **(b)** normal birthweight vs. macrosomia; adjusting for continuous age, sex, survey year, geographic region, urban–rural status, gestational age, childbearing age, and mother’s education; When a categorical adjustment variable duplicates a subgroup factor, it is automatically removed.

**Figure 4:**
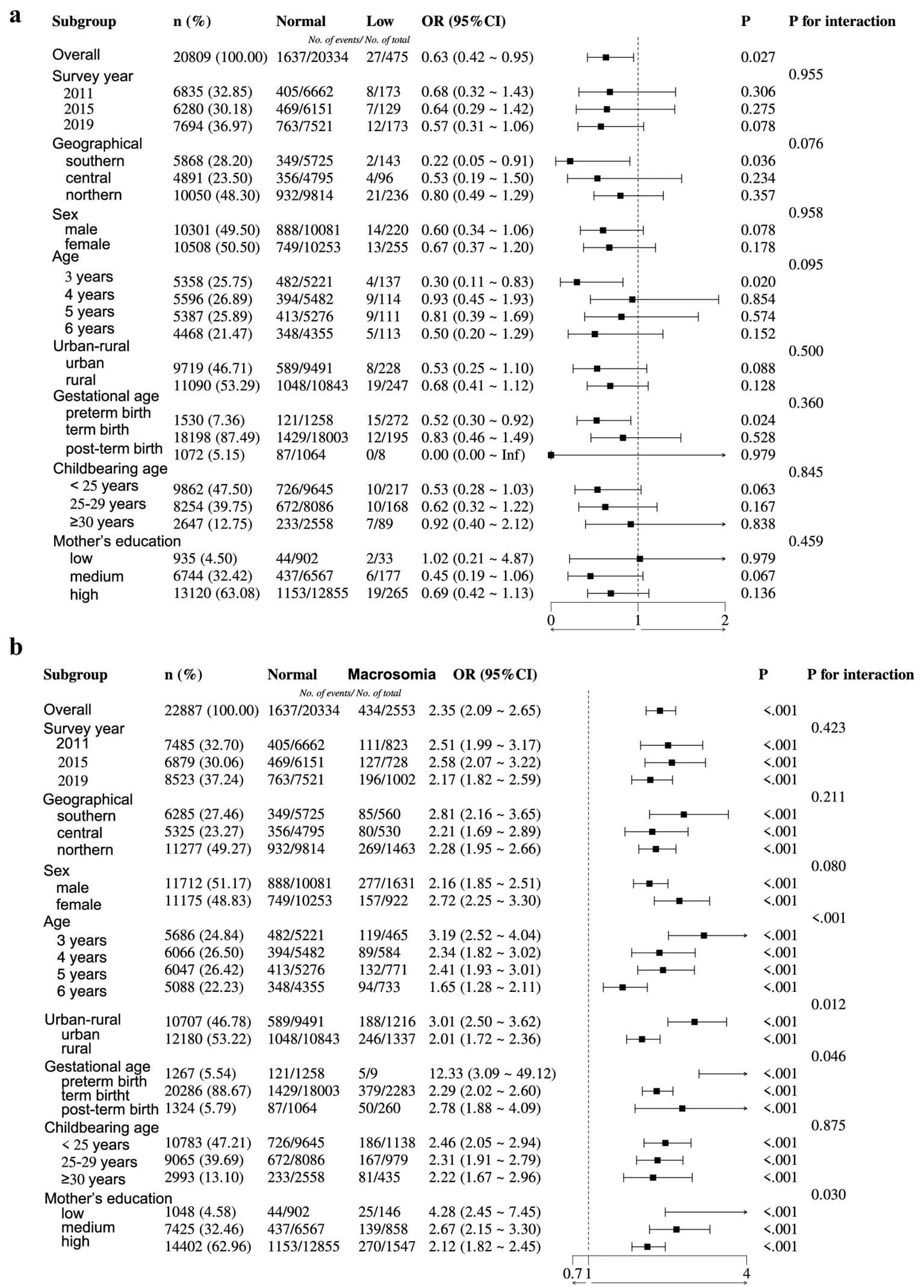
Subgroup analysis of obesity risk for birth weight in children aged 3–6 years. **(a)** Normal birthweight vs. low birthweight; **(b)** normal birthweight vs. macrosomia; adjusting for continuous age, sex, survey year, geographic region, urban–rural status, gestational age, childbearing age, and mother’s education; When a categorical adjustment variable duplicates a subgroup factor, it is automatically removed.

**Figure 5:**
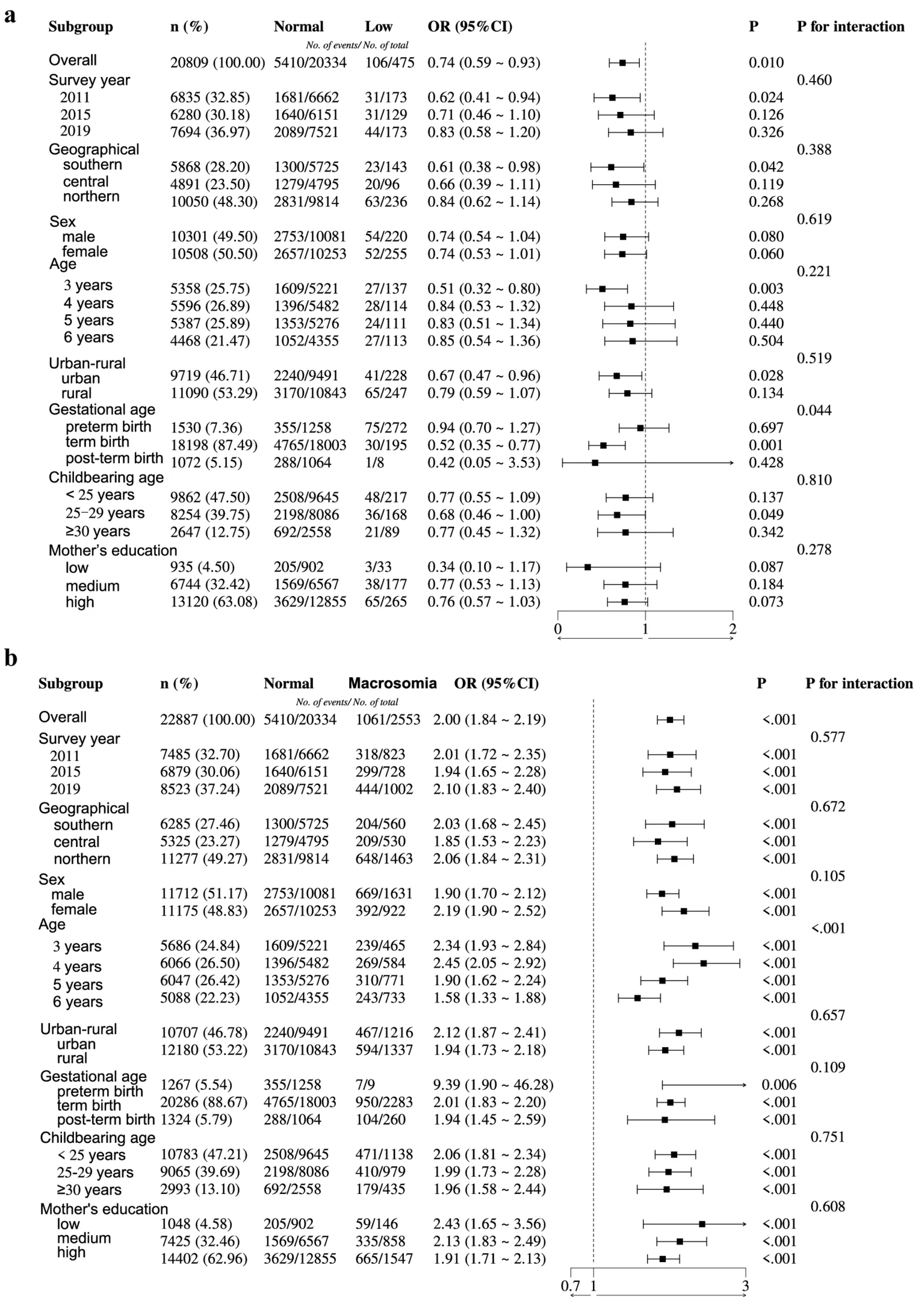
Subgroup analysis of overweight/obesity risk for birth weight in children aged 3–6 years. **(a)** Normal birthweight vs. low birthweight; **(b)** Normal birthweight vs. macrosomia; adjusting for continuous age, sex, survey year, geographic region, urban–rural status, gestational age, childbearing age, and mother’s education; When a categorical adjustment variable duplicates a subgroup factor, it is automatically removed.

Figure 4 shows that although there was a difference in overall obesity risk between low birth weight and normal groups (*p* = 0.027, approaching 0.05), subgroup analysis showed statistical significance only in the southern Jiangsu region, 3-year-old, and preterm birth groups (*p* < 0.05), while obesity risk in all other subgroups was not statistically significant (all *p* > 0.05), and subgroup interactions were not demonstrated. The subgroup analysis results revealed that low birth weight has limited effect on obesity risk. In all subgroups, macrosomia was associated with higher obesity risk (OR: 1.65 to 3.19, all *p* < 0.01), and interactions showed higher obesity risk in macrosomic children of younger age, urban residence, and lower mother’s education (*p* < 0.05).

Figure 5 shows statistically significant overall differences in overweight/obesity risk between low birth weight and normal groups (*p* = 0.010, approaching 0.05). Significant OR values existed only in subgroups of 2011 survey year, southern Jiangsu, 3-year-old children, urban, and term birth (*p* < 0.05); except for gestational age (*p* < 0.05), no significant interactions between subgroups were found (all *p* > 0.05). In all subgroups, macrosomia was associated with higher overweight/obesity risk (OR: 1.58 to 2.45, all *p* < 0.01), and interactions indicated higher risk in younger age groups (*p* < 0.001).

### 3.5 Dose–response relationship between birth weight and overweight and obesity risk

To visually illustrate this relationship, OR curves derived from RCS analysis were used to test the dose–response relationship between birth weight (as a continuous variable) and risks of overweight, obesity, and overweight/obesity in children aged 3–6 years. Figure 6 shows RCS trend analysis with 5 knots (5, 27.5, 50, 72.5, 95%) using a birth weight of 3.4 kg as the reference value. After adjusting for covariates (continuous age, sex, survey year, geographic region, urban–rural status, gestational age, childbearing age, and mother’s education), the risk of obesity and overweight/obesity increased gently in the lower birth weight range (1.5–4 kg) and sharply in the higher range (4–6 kg), demonstrating a nonlinear dose–response relationship (P for nonlinearity < 0.001). The risk of overweight showed a flat trend at both ends and an increase in the middle range, with a relatively weak overall nonlinear dose–response relationship (*p* = 0.021, close to 0.05).

**Figure 6:**
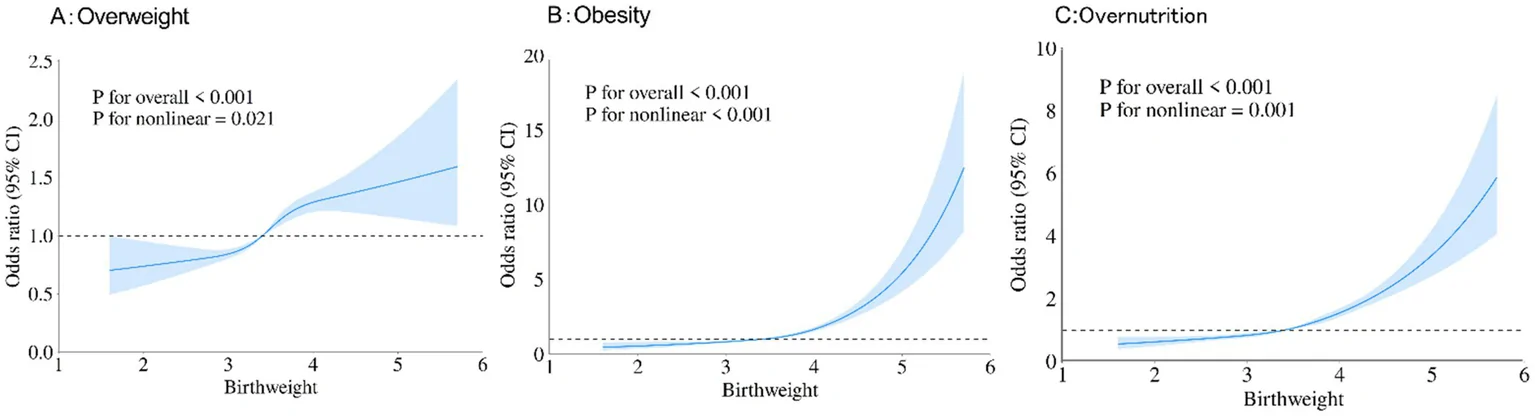
RCS analysis of birth weight with risks of overweight, obesity, and overweight/obesity. Shaded areas represent the 95% confidence intervals for ORs. **(A)** Overweight. **(B)** Obesity. **(C)** Overnutrition.

### 3.6 Sensitivity analysis

To validate the reliability and robustness of results, we employed three sensitivity analysis methods. (1) Mixed-effects logistic regression models with 13 cities as random intercept variance to adjust for clustering effects: Appendix Table A1 shows that after adjusting for clustering effects, OR and *p*-values for overweight, obesity, and overweight/obesity risks for low birth weight or macrosomia groups showed only minimal changes compared with the standard multivariate logistic regression in Table 3, demonstrating high stability of all results. Appendix Table A2 shows that after covariate adjustment, *p*-values ranged from 0.035–0.047, indicating statistically significant clustering effects, but the effect size was small with random intercept variance of 0.038–0.059 and ICC of 0.011–0.018, suggesting that clustering effects accounted for only 1.1–1.8% of the total variance. (2) Sensitivity analysis by changing birth weight category cut-points: the low birth weight cut-point was increased to 2.6 kg, while the macrosomia cut-point was decreased to 3.9 kg, correspondingly increasing the sample sizes of low birth weight and macrosomia groups. Appendix Table A3 shows that the OR for overweight in the low birth weight group changed from 0.834 to 0.757. This change may be attributed to the cut-point adjustment leading to increased overweight risk in the medium birth weight range, which is consistent with the trend shown in the RCS plot (Figure 6); the OR for obesity increased from 0.628 to 0.844, with *p*-values showing no statistical significance, indicating limited effect of low birth weight on obesity; although OR values for overweight, obesity, and overweight/obesity in the macrosomia group decreased, the trend remained unchanged, consistent with RCS plot results. (3) Additionally, we assessed result robustness by excluding gestational age, a potentially key covariate that could influence outcomes. Appendix Table A4 shows that after excluding gestational age, results showed only minimal changes, revealing that gestational age was not a potential confounding factor. Overall, our sensitivity analyses validated the stability of results, and combined with subgroup analysis findings, we can conclude that low birth weight has limited impact on overweight and obesity in children aged 3–6 years.

## 4 Discussion

Over the past four decades, the prevalence of overweight and obesity among Chinese children has surged dramatically. The influencing factors of obesity are multifaceted, encompassing individual genetic and behavioral factors as well as social and environmental drivers (1–3, 32). Our study focused on the effects of birth weight and demographic characteristics related to early childhood life on overweight and obesity in children aged 3–6 years. Data from three survey waves across different years and analyzed potential temporal effects. The main findings are twofold: first, after adjusting for covariates, low birth weight (< 2.5 kg) had limited effects on overweight and obesity in children aged 3–6 years (however, gestational age exhibits heterogeneity, whereby term-born low birth weight children have lower overweight and obesity risk, while preterm infants show no difference), while macrosomia (≥ 4.0 kg) was associated with significantly higher risks of overweight, obesity, and overweight/obesity compared with normal birth weight, with the effect decreasing with age. Second, after covariate adjustment, RCS analysis revealed intuitive nonlinear relationships: birth weight showed nonlinear associations with weight, BMI, and risks of obesity and overweight/obesity at 3–6 years, with a gentle slope in the lower range and a steeper slope in the higher range.

Children with high birth weight have a higher risk of overweight, obesity, and overweight/obesity in childhood, a finding supported by strong evidence; several reviews have also confirmed this strong association (21, 23, 33). A study across 22 countries in the WHO European Region found that higher birth weight (> 4.0 kg) was associated with an increased risk of overweight (34). Other studies on Chinese children have confirmed that high birth weight confers a significantly higher risk of overweight or obesity in children aged 6 months to 3 years (14), 3–6 years (17), and 7–17 years (26, 35). Our findings are consistent with previous research, further validating that Chinese children with high birth weight have a higher obesity risk from infancy to adolescence. Therefore, children with high birth weight (> 4.0 kg) require priority monitoring for overweight/obesity risk during growth, which should persist into adolescence. Potential mechanisms include maternal overweight/obesity increasing fetal obesity risk, intrauterine nutrition being linked to birth weight, and birth weight exerting a substantial impact on childhood obesity development (36–38). Higher insulin-like growth factor levels associated with higher birth weight may also increase obesity risk in children and adults (39).

Discrepancies in previous studies primarily focus on whether low-birth-weight children have a lower risk of overweight and obesity. Several large-cohort studies from Asia and China have reported similar results: high birth weight increases childhood overweight/obesity risk, while low birth weight reduces this risk (14, 17, 35), suggesting that low birth weight may be “protective” against childhood obesity. However, other studies indicate that high birth weight increases childhood obesity risk, whereas low birth weight shows no difference in obesity risk compared with normal birth weight (23–26). Our findings are more aligned with the latter. We conclude that low birth weight (< 2.5 kg) has limited effects on overweight and obesity in children aged 3–6 years; although the ORs for obesity and overweight/obesity were statistically significant, no significant association was observed for overweight risk. When the low birth weight cutoff was increased to 2.6 kg in sensitivity analyses, the OR for obesity risk became non-significant. More importantly, subgroup analysis revealed that after stratification by gender, age, geography, and urban–rural status, obesity risk in almost all low birth weight subgroups showed no statistical significance. The potential reason may be that logistic regression with covariate adjustment after stratification is more precise, which elevated the *p*-values that were originally close to 0.05 in the overall analysis (*p* = 0.027 for low birth weight on obesity, *p* = 0.010 for low birth weight on overweight/obesity) to above 0.05 in each subgroup. Therefore, based on sensitivity and subgroup analyses, we are more confident that low birth weight has limited impact on overweight and obesity in children aged 3–6 years. A noteworthy highlight is that through subgroup analysis of 8 factors, we further identified gestational age as the main source of heterogeneity—term-born low birth weight children have lower overweight and obesity risk, while preterm low birth weight children have similar overweight and obesity risk to normal birth weight children. This means that for the same low birth weight, if caused by malnutrition or other reasons, overweight and obesity risk is very low, whereas if due to preterm birth, overweight and obesity risk remains unreduced. Our finding of gestational age heterogeneity may help explain controversies and inconsistencies in previous studies. These conclusions remind clinicians and parents that overweight/obesity risk still requires attention in children with low birth weight. Parents of low-birth-weight children may provide excessive dietary intake during early childhood, potentially leading to “catch-up” growth (40); the interaction between early childhood catch-up growth and childhood/adolescent obesity warrants further investigation. Additionally, some studies have identified gender as a modifier: a Chinese study reported stronger associations in boys (26), while an Australian analysis found associations in girls (24). Regarding overweight/obesity risk in macrosomia, subgroup analyses detected no significant sex differences, and the associations were not influenced by survey year, north–south geography, urban–rural status, childbearing age and mother’s education. However, there was an interaction with child age, with the association weakening as age increased.

The asymmetrical overweight/obesity risks between high- and low-birth-weight groups suggest a nonlinear relationship between birth weight and BMI/overweight/obesity risk. The RCS plots visually demonstrate this nonlinearity: birth weight exhibits nonlinear associations with weight, BMI, and risks of obesity and overweight/obesity at 3–6 years, with a gentle slope in the lower range and a steeper slope in the higher range. This nonlinear pattern is similar to the RCS plot of birth weight and obesity in children/adolescents aged 7–17 years (26). Our study fills the gap in understanding this nonlinear dose–response trend in children aged 3–6 years.

Strengths of this study include: First, relatively large sample size with representative sampling methods and data covering 13 cities in Jiangsu Province (including rural areas within each administrative region). Second, due to the retrospective analytical approach with temporal sequence, all independent variables preceded the dependent variable occurrence, making the study design similar to a retrospective cohort with minimal loss to follow-up, thus facilitating causal relationship establishment. Third, we incorporated eight covariates. Extending beyond previous studies restricted to age and gender, we examined the potential moderating roles of temporal changes, urban–rural setting, and geographic factors, thereby identifying specific implications for practice. Limitations of this study include several aspects: First, despite being official government national physical fitness surveys with dedicated quality supervisors requiring birth information completion based on birth certificates, it remains difficult to avoid some participants relying on memory for reporting, potentially introducing recall bias/reporting bias. Second, lack of data on additional potential covariates, and data cannot represent all of China but only the economically developed eastern region.

## 5 Conclusion

Birth weight shows nonlinear associations with weight, BMI, and risks of obesity/overweight/obesity in children aged 3–6 years, characterized by a gentle slope in the lower birth weight range and a steeper slope in the higher range. Low birth weight has limited impact on the development of overweight and obesity, but gestational age shows significant heterogeneity; whereas macrosomia confers significantly higher risks of overweight, obesity, and overweight/obesity compared with normal birth weight, with this effect diminishing with increasing age.

## Data Availability

The original contributions presented in the study are included in the article/supplementary material, further inquiries can be directed to the corresponding author.

## Footnotes

1
www.zstats.net
