# Supplementary material for: Nonlinear association between birth weight and overweight/obesity in children aged 3–6 years: a retrospective analysis of 13 cities in China
Source: Front Nutr. 2026 Sep 23;13:1883738. doi: 10.3389/fnut.2026.1883738 (PMC13641986; doi:10.3389/fnut.2026.1883738)
Supplement: Supplementary file 1 [file Data_sheet_1.pdf]

STROBE Statement—Checklist of items that should be included in reports of ***cross-sectional studies***

	Item No	Recommendation	Page
**Title and abstract**	1	(a) Indicate the study's design with a commonly used term in the title or the abstract	Title and abstract
		(*b*) Provide in the abstract an informative and balanced summary of what was done and what was found	Title and abstract
**Introduction**			
Background/rationale	2	Explain the scientific background and rationale for the investigation being reported	1 Introduction
Objectives	3	State specific objectives, including any prespecified hypotheses	1 Introduction
**Methods**			
Study design	4	Present key elements of study design early in the paper	2.1 Study design
Setting	5	Describe the setting, locations, and relevant dates, including periods of recruitment, exposure, follow-up, and data collection	2.2 Participants and Data Collection
Participants	6	(*a*) Give the eligibility criteria, and the sources and methods of selection of participants	2.2 Participants and Data Collection
Variables	7	Clearly define all outcomes, exposures, predictors, potential confounders, and effect modifiers. Give diagnostic criteria, if applicable	2.3Measurements and Definition
Data sources/ measurement	8*	For each variable of interest, give sources of data and details of methods of assessment (measurement). Describe comparability of assessment methods if there is more than one group	2.3Measurements and Definition
Bias	9	Describe any efforts to address potential sources of bias	2.4 Statistical analysis
Study size	10	Explain how the study size was arrived at	2.2 Participants and Data Collection
Quantitative variables	11	Explain how quantitative variables were handled in the analyses. If applicable, describe which groupings were chosen and why	2.3Measurements and Definition
Statistical methods	12	(*a*) Describe all statistical methods, including those used to control for confounding	2.4 Statistical analysis

		(*b*) Describe any methods used to examine subgroups and interactions	2.4 Statistical analysis
		(*c*) Explain how missing data were addressed	2.4 Statistical analysis
		(*d*) If applicable, describe analytical methods taking account of sampling strategy	2.4 Statistical analysis
		(*e*) Describe any sensitivity analyses	2.4 Statistical analysis
**Results**			
Participants	13*	(a) Report numbers of individuals at each stage of study—eg numbers potentially eligible, examined for eligibility, confirmed eligible, included in the study, completing follow-up, and analysed	2.2 Participants and Data Collection
		(b) Give reasons for non-participation at each stage	2.2 Participants and Data Collection
		(c) Consider use of a flow diagram	2.2 Participants and Data Collection
Descriptive data	14*	(a) Give characteristics of study participants (eg demographic, clinical, social) and information on exposures and potential confounders	3.1Participants' Characteristics and Univariate Analysis
		(b) Indicate number of participants with missing data for each variable of interest	3.1Participants' Characteristics and Univariate Analysis
Outcome data	15*	Report numbers of outcome events or summary measures	3.1Participants' Characteristics and Univariate Analysis
Main results	16	(*a*) Give unadjusted estimates and, if applicable, confounder-adjusted estimates and their precision (eg, 95% confidence interval). Make clear which confounders were adjusted for and why they were included	3.4 Birth weight and the risk of overweight and obesity in children aged 3–6 years
		(*b*) Report category boundaries when continuous variables were categorized	3.4 Birth weight and the risk of overweight and obesity in children aged 3–6 years
		(*c*) If relevant, consider translating estimates of relative risk into absolute risk for a meaningful time period	3.3Prevalence of overweight and obesity across different birth weight categorie
Other analyses	17	Report other analyses done—eg analyses of subgroups and interactions, and sensitivity analyses	3.6 Sensitivity Analysis

Discussion

Key results	18	Summarise key results with reference to study objectives	4 Discussion
Limitations	19	Discuss limitations of the study, taking into account sources of potential bias or imprecision. Discuss both direction and magnitude of any potential bias	4 Discussion
Interpretation	20	Give a cautious overall interpretation of results considering objectives, limitations, multiplicity of analyses, results from similar studies, and other relevant evidence	4 Discussion
Generalisability	21	Discuss the generalisability (external validity) of the study results	4 Discussion
**Other information**			
Funding	22	Give the source of funding and the role of the funders for the present study and, if applicable, for the original study on which the present article is based	Funding

*Give information separately for exposed and unexposed groups.

Note: An Explanation and Elaboration article discusses each checklist item and gives methodological background and published examples of transparent reporting. The STROBE checklist is best used in conjunction with this article (freely available on the Web sites of PLoS Medicine at http://www.plosmedicine.org/, Annals of Internal Medicine at http://www.annals.org/, and Epidemiology at http://www.epidem.com/). Information on the STROBE Initiative is available at http://www.strobe-statement.org/.
