# Supplementary material for: Nonlinear association between birth weight and overweight/obesity in children aged 3–6 years: a retrospective analysis of 13 cities in China
Source: Front Nutr. 2026 Sep 23;13:1883738. doi: 10.3389/fnut.2026.1883738 (PMC13641986; doi:10.3389/fnut.2026.1883738)
Supplement: Supplementary file 2 [file Table_1.docx]

Table A1. Sensitivity analysis: Mixed-effects logistic regression models to adjust for clustering effects. (Risks of overweight, obesity, and overweight/obesity associated with low birth weight and macrosomia).

| **Dependent variable** | **Low Birthweight** [**^a^**](javascript:popRef('jah34181-note-0013')) | | **Macrosomia** [**^a^**](javascript:popRef('jah34181-note-0013')) | | **Low Birthweight** [**^b^**](javascript:popRef('jah34181-note-0013')) | | **Macrosomia** [**^b^**](javascript:popRef('jah34181-note-0013')) | |
| --- | --- | --- | --- | --- | --- | --- | --- | --- |
|  | **OR (95% CI)** | ***P*** | **OR (95% CI)** | ***P*** | **OR (95% CI)** | ***P*** | **OR (95% CI)** | ***P*** |
| Overweight | 0.875 (0.685, 1.117) 0.285 | | 1.404 (1.274, 1.547) <0.001 | | 0.831 (0.621, 1.074) 0.157 | | 1.456 (1.318, 1.608) <0.001 | |
| Obesity | 0.675 (0.456, 1.000) 0.050 | | 2.268 (2.020, 2.547) <0.001 | | 0.608 (0.404, 0.914) 0.017 | | 2.317 (2.057, 2.610) <0.001 | |
| Overweight/obesity | 0.785 (0.630, 0.977) 0.030 | | 1.915 (1.758, 2.086) <0.001 | | 0.724 (0.575, 0.912) 0.006 | | 1.994 (1.827, 2.176) <0.001 | |

Note: ^a^, crude model; ^b^, adjusted for continuous age, sex, survey year, geographic region, urban-rural status, gestational age, childbearing age and mother’s education.

Table A2. Sensitivity analysis: Clustering effect magnitude of mixed-effects logistic regression models.

| **Dependent variable** | **Random**  **intercept variance**  **(95% CI)** ^a^ | ***P*** | ***ICC*** | **Random intercept variance**  **(95% CI)** ^b^ | ***P*** | ***ICC*** |
| --- | --- | --- | --- | --- | --- | --- |
| Overweight | 0.047 (0.020, 0.155) | 0.026 | 0.014 | 0.038 (0.014, 0.102) | 0.047 | 0.011 |
| Obesity | 0.068 (0.028, 0.164) | 0.026 | 0.020 | 0.059 (0.022, 0.161) | 0.049 | 0.018 |
| Overweight/obesity | 0.067 (0.029, 0.155) | 0.020 | 0.020 | 0.059 (0.023, 0.151) | 0.035 | 0.018 |

Note: ^a^, crude model; ^b^, adjusted for continuous age, sex, survey year, geographic region, urban-rural status, gestational age, childbearing age and mother’s education.

Table A3. Sensitivity analysis: Changing birth weight category cut-points (Risks of overweight, obesity, and overweight/obesity associated with low birth weight and macrosomia).

| **Dependent**  **variable** | **Low Birthweight** [**^a^**](javascript:popRef('jah34181-note-0013')) | | **Macrosomia** [**^a^**](javascript:popRef('jah34181-note-0013')) | | **Low Birthweight** [**^b^**](javascript:popRef('jah34181-note-0013')) | | **Macrosomia** [**^b^**](javascript:popRef('jah34181-note-0013')) | |
| --- | --- | --- | --- | --- | --- | --- | --- | --- |
|  | **OR (95% CI)** | ***P*** | **OR (95% CI)** | ***P*** | **OR (95% CI)** | ***P*** | **OR (95% CI)** | ***P*** |
| Overweight | 0.789 (0.651, 0.955) 0.015 | | 1.303 (1.200, 1.415) <0.001 | | 0.757 (0.621, 0.923) 0.009 | | 1.317 (1.211, 1.432) <0.001 | |
| Obesity | 0.837 (0.637, 1.102) 0.205 | | 1.871 (1.687, 2.076) <0.001 | | 0.844 (0.637, 1.119) 0.239 | | 1.855 (1.668, 2.063) <0.001 | |
| Overweight/obesity | 0.781 (0.661, 0.922) 0.004 | | 1.616 (1.504, 1.737) <0.001 | | 0.758 (0.638, 0.901) 0.002 | | 1.628 (1.513, 1.752) <0.001 | |

Note: ^a^, crude model; ^b^, adjusted for continuous age, sex, survey year, geographic region, urban-rural status, gestational age, childbearing age and mother’s education.

Table A4. Sensitivity analysis: Excluding gestational age as a potential key covariate (Risks of overweight, obesity, and overweight/obesity associated with low birth weight and macrosomia).

| **Dependent**  **variable** | **Low Birthweight** [**^a^**](javascript:popRef('jah34181-note-0013')) | | **Macrosomia** [**^a^**](javascript:popRef('jah34181-note-0013')) | | **Low Birthweight** [**^b^**](javascript:popRef('jah34181-note-0013')) | | **Macrosomia** [**^b^**](javascript:popRef('jah34181-note-0013')) | |
| --- | --- | --- | --- | --- | --- | --- | --- | --- |
|  | **OR (95% CI)** | ***P*** | **OR (95% CI)** | ***P*** | **OR (95% CI)** | ***P*** | **OR (95% CI)** | ***P*** |
| Overweight | 0.876 (0.686, 1.118) 0.286 | | 1.429 (1.297, 1.574) <0.001 | | 0.867 (0.678, 1.109) 0.257 | | 1.453 (1.317, 1.604) <0.001 | |
| Obesity | 0.688 (0.465, 1.018) 0.062 | | 2.339 (2.085, 2.624) <0.001 | | 0.710 (0.479, 1.053) 0.088 | | 2.324 (2.066, 2.614) <0.001 | |
| Overweight/obesity | 0.792 (0.637, 0.986) 0.037 | | 1.962 (1.803, 2.135) <0.001 | | 0.796 (0.639, 0.993) 0.043 | | 1.989 (1.824, 2.168) <0.001 | |

Note: ^a^, crude model; ^b^, adjusted for continuous age, sex, survey year, geographic region, urban-rural status, gestational age, childbearing age and mother’s education.
